# Educating physicians in seventeenth-century England

**DOI:** 10.1017/S0269889719000188

**Published:** 2019-08-08

**Authors:** Jonathan Barry

**Affiliations:** University of Exeter

**Keywords:** Physicians, apprenticeship, education, Restoration London

## Abstract

The tension between theoretical and practical knowledge was particularly problematic for trainee physicians. Unlike civic apprenticeships in surgery and pharmacy, in early modern England there was no standard procedure for obtaining education in the practical aspects of the physician’s role, a very uncertain process of certification, and little regulation to ensure a suitable reward for their educational investment. For all the emphasis on academic learning and international travel, the majority of provincial physicians returned to practice in their home area, because establishing a practice owed more to networks of kinship, patronage and credit than to formal qualifications. Only when (and where) practitioners had to rely solely on their professional qualification to establish their status as young practitioners that the community could trust would proposals to reform medical education, such as those put forward to address a crisis of medicine in Restoration London, which are examined here, be converted into national regulation of medical education in the early nineteenth century, although these proposals prefigured many informal developments in medical training in the eighteenth century.

## 1. Introduction

The tension between minds and hands (or theoretical and practical knowledge) was particularly problematic for trainee physicians in seventeenth-century England. In modern times, professional education offers a standard and often subsidized means for obtaining both theoretical and practical knowledge, which is then certified and rewarded with privileged access to certain occupations (Bonner 2000). One reason for this is recognition that “the market” would struggle, both to finance a long and uncertain training and to deliver rewards for talent, given that “the public” are not judged capable of deciding whether the medical practice then offered was of the correct standard, nor is it acceptable for failures of practice to drive out the unacceptably bad. But in early modern times, in England at least, there was no standard procedure (except for urban apprenticeships in surgery and pharmacy, not physic) for obtaining the correct medical education, nor funding except from family resources, a very uncertain process of certification, and a mixed level of regulation to ensure any kind of protection for those then offering their medical services in competition with others without such qualifications to ensure that they reaped a suitable reward for their educational investment.^1^

Furthermore, the only recognized educational path for the physician was through university training. This performed the key role of distinguishing the physician as a man of learning, whose mental capacities had been developed by knowledge of the traditional authorities and ability to analyze cases logically, so placing him (supposedly) above other types of healer who relied on hands-on knowledge and experience. However, it did not, in itself, prepare the student for the practical aspects, either of treating the sick or running a business as a medical practitioner. Furthermore, as books containing medical knowledge became widely available, including in the vernacular, and as new experimental knowledge in areas such as anatomy and pharmacy were given increased importance, university-trained physicians faced major challenges to their claims to be the best-trained medical practitioners. Yet, in England at least, there was no major overhaul of medical education to address this challenge until the nineteenth century, although piecemeal changes in the eighteenth century, associated with new hospital training, are generally credited with providing a new model in practice ahead of formal changes. This article considers a neglected series of proposals for reform in the Restoration period, designed to address precisely the question of how young physicians might obtain practical experience, which arose from the disputes in London about who was best qualified to give medical care. These proposals foreshadowed many of the later developments in terms of skills training, but the proposed Restoration model of collegial partnership controlled by physicians themselves was not followed in the later period.

## 2. Training for a Medical Career

Establishing how physicians were educated is no easy task, because (for England at least) we have very few personal statements reporting on their medical training, and those we have are usually from the most educated, who either reveal details of their education in their publications (often polemically, to demonstrate that they are better educated than their critics) or have well-studied careers.^2^ These elite examples reinforce the other feature of the existing research, which is that it largely derives from two linked themes, namely the histories of the most prestigious medical universities, and the *peregrinatio* which medical practitioners took around western Europe moving between these universities. We have substantial information about enrollment at Bologna and Padua, Paris and Montpellier, Leiden and Utrecht, and various German-speaking universities, including British students (Grell et al. 2010). Significantly, Oxford and Cambridge were not featured on the international medical circuit, nor were the Scottish universities before 1726, when Edinburgh gradually joined the international scene (Lawrence 2006).

Those accounts we have in English of the “ideal medical education” do indeed describe the same peregrination, ideally taking in three or four of these universities, as well as other humanistic and medical highlights in Italy, France, the Low Countries, and Germany.^3^ But it is unlikely that more than a small percentage even of the graduate physicians of early modern England completed this highly time-consuming and expensive operation; establishing what average physicians did is much harder. Even in the case of apprenticeships for surgeons and apothecaries, where we have extensive quantitative material available, we have almost no qualitative evidence, either for the nature of the training offered, nor for how individuals experienced that education.

Here I shall be tackling this question qualitatively not quantitatively, and not from personal papers and individual career studies, but rather indirectly by considering what one can learn about medical education from the published medical debates of the later seventeenth century. Controversies over the boundaries of medical practice among and between physicians, surgeons, and apothecaries (and of all these against other practitioners) led to some interesting (if unrealized) proposals about medical education, which sought to fuse learning with clinical experience and a form of apprenticeship, to produce physicians with “experimental” knowledge. Intriguingly, these presaged key developments in medical education in the eighteenth and nineteenth centuries.

Returning to the demands for knowledge, writers repeatedly stressed that the tag *ars longa, vita brevis* applied nowhere more starkly than in medicine, where the level of knowledge acquisition required for successful practice was potentially infinite: one sarcastic critique of medical learning (by an M.D. of long experience) suggested that “if reading and study, without instruction and practice were the best and only method of attaining the highest level of knowledge in physic, … at least fifty or sixty years studying in the university” was required (Chamberlen 1694, 60). Defenders of academic physic considered that Oxford and Cambridge statutes (anticipating 12 years study before obtaining an M.D.) were realistically measuring what was required. The physician was expected to be a master of arts and of both natural and human philosophy as well as an expert in both surgery and pharmacy since, within the standard model of the profession, he (and of course it was “he”) was expected to oversee the work of surgeons (external and manual medicine) and apothecaries (preparation of medicines) (Axtell 1970). The body of learning required to be mastered expanded rapidly through the early modern period as knowledge in natural history, chemistry, and anatomy multiplied, and as it became expected that obtaining such knowledge required experimental work, not merely learning from authoritative texts. These changes highlighted the growing artificiality of the divisions of practice between physicians, surgeons, and apothecaries. Some authors claimed that the medical profession was inherently a single one, both originally when the ancient authorities had written (Galen had been a surgeon, and Hippocrates was a protean figure) and because modern developments had made a comprehensive approach to medicine essential. Moreover, physicians were increasingly justifying their own status in terms of expertise which might, traditionally, have been seen as lying within the expertise of the surgeon or pharmacist, namely anatomy and the production of medicines, with their strong manual components (Cook 1989, 1990b).

Yet this knowledge acquisition was merely the prolegomena to the crucial measure of success, namely its application to the practice of medicine, as measured by the health and curing of patients. This practice itself was complex, as the physician had to learn to diagnose illness, prognosticate the outcomes and, where appropriate, apply suitable therapies, as well as recommend preventative measures (particularly diet and exercise) for the preservation of health. This was within a common understanding that both diseases and their therapies existed not as independent entities measurable by distinct characteristics, but as complex manifestations of bodily imbalance whose signs (and effects) would vary according to the age, sex, humoral temperament, climate, life style, and other features of the patient and the environment (Cook 1994, 2011; Wear 2000).

If being able to solve all these manifold problems was, in theory, primarily the duty of the physician using his logical skills and fund of theoretical knowledge, in practice both surgeons and apothecaries found themselves required to perform the same functions: the relatively small number of physicians based mostly in towns meant that rural populations, plus those on ships or in armies, and many poorer people who could not afford a physician, sought medical advice from the much larger number of surgeons (especially in war and at sea) or apothecaries, especially in England where there were no state regulations limiting their numbers, and only weak state support for laws limiting giving medical advice to licensed physicians. Indeed, such was the unmet demand for medical advice that it was constantly given by many other people, such as family members, the clergy, charitable gentlewomen, and a host of unregulated healers, including cunning people and “empirical practitioners” of all sorts whose claims rested on their reputed practical efficacy, as “operators” using manual skills or vendors of secret remedies, rather than on educational or training qualifications (Pelling and Webster 1979; Jenner and Wallis 2007). As they constantly complained, physicians in general found it almost impossible to persuade the public to respect their claim to a distinctive expertise in providing medical advice and care, and the newly-trained physician must have found the task even more daunting.

Faced with these challenges, it has been shown that even graduate physicians born in the second half of the seventeenth century (and many people who called themselves, or were called by others, physicians, did *not* have a medical degree) displayed a strong tendency to set up practice in or close to the place where they were brought up. The only significant exceptions were those who moved to practice in London or its environs, or stayed close to Oxford or Cambridge. Graduates of overseas universities were slightly more likely to be mobile (especially to practice in London) than those educated at Oxford or Cambridge, but even these generally moved back to their home area. Once established in practice, their most likely further move (if any) was to the nearest large town. Andrea Davies, whose research has established this pattern (which changes during the eighteenth century), concludes that the physicians required local networks of family, friends, and professional ties to establish the necessary social basis on which to practice. In many cases, either during their university education (which did not require continual attendance, especially in the later stages) or immediately after it, they returned to their home area to practice under the wing of an established practitioner: a significant minority were the sons or relatives of practitioners and may have worked with them, eventually taking over their practice. But even where this was not the case, the chief requirement to establish a medical practice was to win the trust of the local community, and this depended less on one’s medical qualifications than on having an effective network of social connections. Even one’s qualifications also had a strong local dimension, because there were very clear regional patterns of attendance at Oxford (the west and most of the midlands) and Cambridge (the east, south-east and north), and even at specific colleges, as well as regional variations in travel overseas for study, so graduates of particular places tended to cluster together, with only London a genuine melting-pot enriched by a substantial proportion of overseas-born practitioners (Davies 2008, chaps. 2-4; cf. Barry 1987; Harley 1994). We know far more about medical rivalries and career patterns in London than we do in the provinces, but it is likely that the London position, where relatively few physicians could rely on kinship and community connections, is highly untypical, which goes far to explain why rivalries in London were so intense.

Davies’ findings for provincial physicians go far to lessen, in practice, the apparent contrast between civic apprenticeships in pharmacy and surgery and the university-based education of physicians. If the latter depended heavily on establishing themselves in a specific community, then they shared this with the apprentice in pharmacy or surgery, who was not simply learning a set of technical skills, but also building his position in a specific town (or its immediate hinterland), with the opportunity to become a freeman of that town and either establish his own business or become the partner/successor of his master. As Patrick Wallis discusses in his article in this issue, the focus of regulation and descriptions of apprenticeship is not on the transmission of specific knowledge, but rather on learning the practice of the trade in that particular place, and establishing networks of trust with potential customers (including which customers could be trusted with credit). How to run a business was probably as critical to success as particular technical skills (Chamberland 2013; Pelling 1994 and 1998; Wallis 2007). Although not all apprentices set up in the town of their training, evidence from Bristol suggests that the others most likely either went back to their place of origin (usually, except in London, the nearby counties) or broke free of their local ties by taking their chances in London, at sea, or in the armed forces. Furthermore, many non-graduates who became recognized as physicians were surgeons or apothecaries who gradually over their careers supplemented their apprenticeship by establishing a local reputation as general practitioners, including physic: they often confirmed this reputation by seeking an episcopal license, with supportive testimonies both from other practitioners and influential local people (Mortimer 2004). In the eighteenth century, especially once degrees became available from the Scottish universities, this practice extended greatly (Burnby 1995; Digby 1994; Loudon 1986; Lane 1985, 1988, 1996). Establishing oneself as a physician was, in short, a process of “life-long learning,” rooted in most cases in specific communities of practice, rather than an intensive and distinct phase of academic learning. However, this left open the question of how the recently graduated physician could establish himself in practice, especially if he sought to operate in London, where community ties would be weakest and competition fiercest, and hence it was in London that the first explicit debate on this subject occurred.

## 3. The Crisis in London Medicine

This article will explore a debate in Restoration London, which, uniquely for England in this period, generated a series of publications that discussed, if briefly, how physicians might obtain better practical experience to help establish their careers.^4^ The debate arose from a crisis in London medicine. Since the educational implications in which we are interested are discussed as an incidental (and relatively minor) aspect of this wider crisis, it is necessary to sketch the context to make sense of its educational aspects. There was an ongoing struggle in Restoration London between at least six groups: the London College of Physicians; a rival group of chemical physicians who sought recognition for a Society of Chemical Physicians; other physicians who were not members of the College; the Society of Apothecaries; the Company of Barber-Surgeons; and the burgeoning population of “empirics” offering their medicines and skills to the public (Cook 1986, 1987 and 1989; Mauck 2012; Pelling 2003).

Although it had many cross-currents, central to this conflict was the question of the role of medicines in medicine, especially a growing demand for “medicines” to become precise and specific commodities associated with curing specific disorders. The growth of public demand for “medicines,” and especially medicines for specific diseases (including the demand of military leaders for standard products for their forces) challenged Galenic medicine which was structured to provide individualized health care and treatment to the better off through the guidance of the physician (Cook 1986, 1994, and 2011; Wear 2000). The attack on Galenism had been led by the champions of chemical physic, who focused on the discovery and production of medicines. This stimulated the expansion of a commercial market for medicines, especially ones not made on demand in an apothecary’s shop (to meet the specific prescription of a specific physician for a specific patient in a specific illness) but pre-produced and even sold as branded items. Supporting factors were a global trade in imported drugs and the impact of printing, allowing labels and directions for products to be produced and products advertised both in handbills and in books and periodicals (Cook 1990a; Wallis and Haycock 2005; Wallis 2006, 2008, and 2012).

Physicians even promoted this tendency, by using printed pharmacopoeia as a means of establishing their control, as they hoped, over apothecaries. Many humanist physicians wished to use their learning to establish the “correct” version of ancient medicines or to judge how best to apply newly discovered drugs or plants. As they struggled to contain the competition from chemical and empirical medicines, they sought to identify an authorized medical armory. Published in Latin, and with its contents limited to the correct composition of each medicine (not a statement of what they should be used for), such pharmacopoeias were acceptable to orthodox physicians. But as part of the radical attack on monopolies of learning in the 1640s and 1650s, Culpeper translated the *London Pharmacopoeia* and spelled out which medicines should be used with specific illnesses **(** Cowen 2001; Furdell 2002; McCarl 1996). More generally, as apothecaries and other critics of physicians asked, why did the public need physicians if they had standard medicines – surely patients, and certainly apothecaries who regularly dispensed them, could usually recognize the illness and prescribe the standard remedy? And if physic comprised applying standard medicines whose ingredients were now public knowledge, then surely that knowledge could be attained by an appropriate mixture of apprenticeship and book learning, and were not apothecaries the most obvious candidates to be general practitioners? The more physicians tried to tighten their control over apothecaries to prevent them from “practicing physic” – that is diagnosing illness and prescribing medicines – the more they encouraged the apothecaries to adopt this argument to garner public support (Levitin 2015).

Amidst an increasingly acrimonious public debate, one line of argument that emerged envisaged a radical new model for physician leadership within a world of medicines. This proposed that the proper and most important task of the physician was to experiment and perfect his own medicines and offer these directly to the patient, not send the patient with a prescription to the apothecary. This drew on Baconianism, as developed during the civil war and then promoted by the Royal Society of London and other “virtuosi” after 1660, with its ideology of experimentation and the pursuit of “useful knowledge” over mere “words” (Cook 1990b). The physician could thus re-establish his claim to authority, but only by demonstrating that his education and mode of practice was not built simply on “words” (or received knowledge), but on his capability to experiment and develop new and useful knowledge, not least in the production of new medicines, so distinguishing his skills from those of the apothecary who was only trained to produce medicines to order. Crucially for our purposes, some advocates of this model (and those who debated with them) proposed a new form of education to fit the physician for this task, and in doing so they threw interesting light on what they identified as the requisite features of a proper medical training.

## 4. The Need for Reform of Medical Education

Education mattered within the debate highlighted in the previous section, because those advocating this approach had to show how such an experimental physician differed from an empiric, “quacking” his own medicines for sordid personal gain and without regulation. To avoid such accusations, those proposing this solution had to stress professional solidarity and openness: competition between such physicians would be a healthy process of offering better remedies to patients and would take place within a collegiate climate, overseen by the College of Physicians. There was also a pressing need for collegiality because the College’s physicians were deeply divided about how best to uphold their interests, partly reflecting their religious and political divisions inherited from the civil war and interregnum (Birken 1983; Cook 1986; Webster 1967). These also inflamed a longstanding tension between graduate physicians with degrees from Oxford and Cambridge, and those with continental degrees. There were good reasons (in the English context) for arguing the superiority of either of these over the other, and also easily available arguments for questioning the value of either (or both). Oxford and Cambridge degrees could be criticized as purely scholastic learning without practical experience, but others complained that many had obtained their Oxford and Cambridge degrees not by following the prescribed study there, but by incorporation of an overseas degree or following a request from royalty or leading patrons. Equally, while some European degrees resulted from substantial periods of study and the submission of a dissertation or examined disputations, other degrees could be obtained (even from leading universities like Leiden) by a short visit and certification of expertise (or, as critics put it, by simply paying a fee). To make matters more complex, many students who had studied at some length at one of the more prestigious but expensive universities, like Paris, then obtained their degree quickly from another, cheaper one. It was all too easy for disgruntled physicians to claim that *their* qualifications were actually superior to those accepted into, or in higher positions within, the London College of Physicians.

Moreover, there was a perceived need to address longstanding weaknesses in the provision of medical education within England, especially now that the prestige of English medicine was finally becoming well-established within European circles, as it had not been (a few stars like Linacre and Caius excepted) until William Harvey. The fact that Oxford and Cambridge were (in European terms) small towns lacking any significance except as universities and with no wealthy clientele to support many practicing physicians, with no hospitals and not even a steady supply of dissectible bodies, meant that they could not match the facilities for medical study offered by the leading European universities. Since the 1640s, individuals, and informal groups around them, especially at Oxford, had begun to pioneer new experimental methods in anatomy, chemistry, and physiology, but although these scientific discoveries were used to defend the credentials of the two universities and their graduates, there was no systematic integration of these developments into the medical curriculum (Allen 1946; Axtell 1970; Bill 1988; Frank 1973; Frank 1997; Lewis 1977; Rook 1969; Rook 1973; Webster 1975). We easily assume that it was the academic prestige of their leading professors which made the top European universities so attractive, but student behavior at places like Padua and Paris suggests that what students valued most was access to clinical and anatomical experience (provided by hospitals and regular dissections), and the ancillary facilities provided by physic gardens, bookshops, and a penumbra of teaching and working opportunities with surgeons, apothecaries, and chemists (as Edinburgh was to exploit so successfully after 1726). These allowed students at these places to obtain the best of both worlds – both theory and practice, reason and experience, prestigious academic qualifications and direct induction to medical practice under the guidance of leading practitioners (Grell et al. 2010, especially Klestinec 2010; Stolberg 2014). The only city in England with a population, elite presence, hospital beds, and a supply of bodies which could support a body of medical practitioners capable of delivering this diverse range of experience was London, but the absence of a university had prevented this from happening.

As Charles Webster has shown, the new demands and opportunities for change created by the civil war and interregnum saw plans for educational reform in medicine, many led by the Hartlib Circle. In 1648, William Petty proposed a teaching hospital or *Nosocomium Academicum*, possibly to be created from one of the existing London hospitals, with a physician to conduct the education of students (who were to have 5 or 6 years of university education already), who would each receive £25 salary to assist the surgeon (who would teach them anatomy), the apothecary and even the nurses and to act as amanuenses to the physician who would direct their reading and training in “herbs, drugs, compound medicines, anatomy, chirurgical instruments, bandages, operations etc.” The surgeon and apothecary would also each have a “mate” and an apprentice. When the medical students had served five years, and the mates four years, the *Nosocomium* would give them a certificate giving permission to practice in any locality. Although Petty’s proposal was not implemented, developments in London hospitals may have involved medical training, especially at the two military hospitals at Ely House and the Savoy, where physicians like John French and the apothecary James Rand supported educational reform, and dissections were regularly performed. Although these two hospitals were abolished at the Restoration, staff at St. Bartholomews were taking private students by 1662 (Webster 1975, 293-300).

Webster also notes the 1656 proposal to create a College of Graduate Physicians in London, which would offer a collegiate setting and support for unlicensed graduate physicians from any university against the monopoly claims of the London College. This was proposed by William Rand (a Leiden graduate), son of the influential apothecary James Rand and a supporter of Helmontian medicine, but it was not intended solely for chemical physicians. Although there is no explicit reference to an educational role for the College, Rand argued that “joint counsel of such a companie” would encourage the advancement “not only of physic, but of all natural discoveries” and, in advocating that there was room for two colleges in London, drew a comparison with the three Inns of Court (which were training centers for lawyers) and pointed to the “spaciousness of London,” predicting “it may be an induction to the establishment of a third universitie.” Webster has described the College of Physicians’ reactions to this proposal, by attempting to portray itself “promoting experimental science while acting as faithful guardian to a system of classical medicine” (ibid., 300-23, 533-4), and the proposals we will now consider belong to this effort.

## 5. The Proposals for Reform

It was in the context of these reform ideas from the 1650s, plus the institutional conflicts of the Restoration period, that the authors of the reform proposals regarding experimental medicine sought to establish the College of Physicians and its membership as the setting for a post-university vocational training for physicians. In the proposals quoted below, they argued for the College to oversee a system in which, while book learning at university remained important, the key stage would be a clinical placement (in modern parlance) in which junior physicians would learn from their seniors, both in their regular practice and (in some versions) also in London’s hospitals. This would provide them with the necessary period of varied experience and collegial guidance before they established their own practices, whether in London or the provinces, and also create a learning environment in which physicians would experiment and create new knowledge as well as passing on existing experience.

The first discussion occurs in the volume usually regarded as launching the reform proposals, namely *A letter concerning the present state of physick, and the regulation of the practice of it in this kingdom written to a doctor here in London* (1665) by T.M.^5^ He introduces his subject by noting that the “unhappy estate of the profession of Physick” was of concern not just of itself but because “it is an honourable way, wherein all the *Gentlemen* of *England* have been ever accustomed to breed and educate some of their Children,” and reform was required so it “may be a decent and worthy means of *subsistence*, as it hath hitherto been, to persons of *ingenuous Birth* and *liberal Education” (*T.M. 1665, 4). He regrets that false principles have meant that “instead of the true way of the *Ancients*, of educating Youth in exercises of *Anatomy*, visiting the Sick with their Masters, examining the nature of Simples in Fields and Gardens, practising to compound Medicines with their own hands; they are bred onely to *Disputation* and the fond Controversies of Books” (ibid., 13). To address this “I look upon your learned *Colledge of Physicians* as the onely company of men in this Kingdom, who are in a just capacity of advancing this good design” (ibid., 14). He proposes that the College ban its members from prescribing through apothecaries, instead preparing their own medicines to be dispensed “by the Physician himself, or his Servant, (or some young Student, educated under him for that and all other things appertaining to his Art)” (ibid., 21). This would “restore the ancient, true, and only fit way of *breeding up young Students* in this Faculty: That is to say, in exercises of *Anatomy*, knowledge of *Herbs*, mixing and compounding *Medicines*, visiting the Sick under the *direction* of a grave *Physician;* not as they are now for the most part, in *speculative* discourses only, and reading of Books” (ibid., 30). Here, despite retaining a socially conservative emphasis on physic as a calling for gentlemen under the control of the College, we see the case clearly made for a broader, experiential training, rather than traditional university learning, as the key educational requirement for effective practice.

To justify this, the author appealed to precedent:

Thus was the late famous Dr. *Wright* the younger educated under Dr. *Fox*, and was the first Physician that dissected at the Colledge, which till his time had ever made use of *Chirurgeons* in their *publick Theatre*. And while the young Physician employs his industry in such services as these for the elder, he gains, (besides what is learn’t from Books and Authors) the long experience of the other, *sees his Patients, hears him discourse of their several cases, considers the Medicines provided for them, and observes their several effects:* All which advantages you now in vain give away to *Apothecaries*, to whom the *Practice of Physick* does not belong. And if this has been the course that all mankinde has ever taken to raise and propagate *Practical* Arts and Trades of daily use in humane life, why should it not be us’d in Physick, which is a *Practical Art* of so much greater consequence? (T.M. 1665, 30-1)

In short, he was recommending an apprenticeship model (as used by apothecaries):

These things consider’d, if a person of three or four years standing in either of our *Universities* (for none else should be entertained by any) should agree with a Physician for a reasonable consideration and acknowledgement to be made (which was held very honourable in *Hippocrates* age, as appears in his Oath, and was the practice at that time) to live with him till he be *Doctor in that Faculty* (being oblig’d to take his *Degrees* in due time with performance of all the accustomed *Exercises:* nor to be admitted to them without the *Certificate* of the said Physician, both concerning his *Time* and good *Deportment*.) Or if a person already *Batchelor in Physick* should contract to stay till he become *Doctor*, taking his *Degrees*, as was before exprest: and these two absolutely prohibited *Practice* till they have taken the degree of *Doctor:* Or if any already *Doctor* should contract for as long a time as he and an elder Physician can agree (not to *Practise* during his continuance with him, without his knowledge and consent) … it were the best and most desireable way to institute men for that Profession, and might with great facility be brought into practice, and is at this time the use of many parts of *Italy*. Nor does a Physician run half the fortune of other men who breed up Youth to their Trades, since the old Physician will ever be held by the world for the more able man. (Ibid., 31-3).

Once again, a conservative gloss is given to a radical proposal by stressing both its classical and Italian precedents and its compatibility with the continued authority of the older physicians, while a (shortened) university background is still presented as essential.

Significantly, Nathaniel Hodges’s response, while not supporting the plan for physicians to dispense their own medicines, echoed T.M.’s view on training as well as his rhetorical strategies for legitimizing such changes:

As in other *Corporations* great care is taken for the *education* of *Apprentices* to their several *Trades*, so a *Collegiate way* herein may be more profitable, and I might hence take a fit occasion to recommend the *practice* of the *Ancients*, who undertook the *tutorage* of *young Students* in *Physick*, which *laudable practice* is still continued in some *Countreys*, and helps more in the *Profession* of *Physick*, then the bare turning over of *Voluminous Authors …* the *Junior Physicians* (I say) being after this manner *initiated*, can more *safely fight* under such *Conduct* against the *desperatest Diseases*, and the *Seniors* will be forward to *transplant* their *abilities*, and even *immortalize* themselves in the continued *Series* of their *Successors*. (Hodges 1666, 91)

Hodges also added another educational argument, attacking apothecaries who practiced physic for failing in their own duties as masters of their apprentices, since they would prioritize visiting patients rather than “to spend their time in *teaching* (according to their engagement) their Servants the Art [i.e. preparation of medicines] which they must be made free to exercise” and so “incapacitate themselves for both” (ibid., 62).

Dr. C. T. (identified by Wing as Christopher Terne but more recently as Timothy Clarke) took the proposal further, first elaborating the issues raised by T.M. and Hodges, and then proceeding to develop a case for hospital-based clinical training. He lamented that

besides the labour of studie, and the expence of as much time, as would make up two Apprentiships, before a man can acquire a lawful title to Physick; the charge of his Education, the taking Degrees, most commonly too his Travel, the making of Experiments, and his Library, may amount to a greater stock, than is necessary to make a man a Master in any other profession whatsoever; and yet there are farr greater encouragments for other professions than for ours. (C.T. 1670, 40)

He also, like Hodges, criticized the training methods of the apothecaries: “if serving an Apprentiship seven years with an *Apothecary*, and reading a little in those three ancient Authors *Frambesarius, Riverius and Primrose*, qualifies any man sufficiently for the practice of Physick, to what end this charge and trouble of taking degrees, this twelve years study before a man can be a Doctor” (ibid., 7). Moreover, he claimed,

divers M[aste]rs amongst them [the apothecaries] of name; that appear full of business, … are wholly ignorant, in those first and simple preparations of infusions: and others … have been forced after they have set up for themselves, to take Journeymen, to teach them how to make up the common medicines of the *Dispensatory*, their Mrs. not having the will, or leasure (to say no other) to teach it them, in the whole time of their Apprentiships. (Ibid., 11)

Yet the apothecaries plotted “to rob both our Universities, of the honour and advantage, of educating generous, and ingenious young men, to the faculty of Physick, nor of conferring upon them honourable degrees, as rewards of their industry and merit” (ibid., 13). He goes on to defend both the training and scientific practices of “College doctors” by invoking the educational peregrination discussed above, noting that they have “been Travellers, and not only seen the best Elaboratories, but have conversed with, and been Disciples to the most celebrated Chymical Operators, our Western part of the world knoweth; we have visited it’s most famous Hospitals, followed and observed the practice of the most exercised Physitians in them” (ibid.).

But by implication, such education by travel signified the lack of appropriate educational opportunities in England. Marshalling evidence “that the *Colledg of Physitians*, have without a Monitor, put themselves into the ways of improving the usefulness of their profession” he then admitted this had been achieved

by the private endeavours of several members of it, our Colledg having none of the advantages of publique Professors, competently endowed from the Publique, for teaching in the several parts of Medicine (onely one endowed Lecture from a private Benefactor for Anatomy), nor no publique Physick garden, nor Elaboratories for Chymical operations, except those of private erection.

But if so much had been achieved without these advantages, what could the College achieve with “those helps”? In particular, if

the Physitians of those Hospitals we have, had such a Salary, as might oblige them to attend those places diligently, and to suffer our younger Physitians for some time to attend on them; by their direction to serve, and minister diligently about the sick, keeping exact accounts of all Accidents, Cures and miscarriages, and of the Anatomie of morbid bodies that die, and such of those as appeared really useful, to be registred in the Archives of our Colledg, might we not hope in some time, to arrive at as much perfection in our Art, as is to be hoped for? (Ibid., 30-31)

He developed the point regarding hospitals, implicitly criticizing T.M.’s focus on learning through the private practice of an individual physician:

the better breeding up of young Students in our faculty, will hardly ever be well acquired, as to the curing of diseases, but by some such constitution for the *Physitians of our Hospitals*, as I have formerly intimated: for although every Doctor in Physick should be able to teach the *Anatomical, Botanical*, and *Pharmacutical* knowledge of our profession, yet there are not many that have so much practice in great Citties as to need help; besides that those that are able to send for *Physitians*, will not alwayes be contented that a younger *Physitian* should be trusted with the knowledge of their infirmities. I could urge a great many inconveniences against this way, but there can be none against constituting our Hospitalls so, that the sick in them might be much better attended, and our younger *Physitians*, in a few Years, acquire great knowledge, in the practicall part of their profession, for thus a three, or four Years attendance, on persons of all ages, and sexes, and sick of all, perhaps of more than all the now known diseases, and under a well experienced practitioner, must give men of any capacity, especially if before but reasonably well grounded, in the true principles of their profession. (Ibid., 38)

Eighteenth-century developments based on hospitals rather than individual practice suggest that C. T. judged this matter better than T.M.

The anonymous author (“A Lover of Truth and the Good of Mankind”) of *An essay for the regulation of the practice of physick* in 1673 developed similar proposals, beginning by comparing the College favourably with the universities as a site for education:

Though the Universities have frequent Physick Exercises, the best Libraries, solemn Dissections, a good Physick Garden, and a few eminent Practitioners, yet though these be allowed, as good preparatory helps to bring a Physician into the world, to qualifie and initiate him for Practise (especially being first admitted to the society of other long experienced Physicians, together with a view and attendance upon their Practice, a thing much wanting and very desirable in *England*, as of singular use both to young Physicians and to the Sick;) yet as to the great propagations and improvements of the Art, there is absolutely necessary (besides long experience) a union, incorporation, frequent association and communication, which, as it is wanting in the Universities, so is it well established in the Colledg of *London*. (“Lover” 1673, 16)

He was clearly aware of the pattern Davies has identified, of graduates returning to their home areas to practice after university, and criticized it, proposing the need for a period of experience in London (or its equivalent), following the training process described by the others. It was

much better, that beginners in Physick should betake themselves to the great Cities, and acquaint themselves with antient Physicians of good note, that they may be guided by them in their Studies, frequent the Shops of Druggists and Apothecaries, see the Practice of Hospitals, be frequent in Dissections and constant at all publick Anatomies, be admitted to the visits of the most eminent Physicians; which course is much better, and safer for the Sick, than that they should settle in little Towns or in the Countrey Villages, and venture upon Practice, upon their own little stock of Knowledg, to the great hazard of the Sick, and the improbability of ever gaining good and justifiable Skill in the Art they profess. (Ibid., 26)

Finally Charles Goodall, who was authorized to defend the College’s legal privileges, returned in 1676 to the potential of using the hospitals, expressing the hope that

seeing our own Academies have no such publick Hospitals amongst them, that his Majesties Colledge of Physicians would propose a method for obtaining some such laudable custome … For doubtless this would not only conduce to the greater improvement of our Art by uniting the Theoretick and Practick part of physick so advantageously together, that the Students thereof, whilst they are diligently pursuing the one, might not miss of obtaining the other; having daily so many and great observations afforded them of the treatment and cure of most acute and chronical distempers … if (having obtained the approbation and consent of the Governors of our publick Hospitals) they would successively take their turns to dissect the bodies of those that dyed of several diseases therein; and diligently observe not only the different morbid impressions, that were made on the several *viscera* and habits of body, in those that dyed of one and the same disease; but likewise of those that dyed of distinct distempers? (Goodall 1676, 59-61)

Through such an arrangement

our own Universities and City of *London* … might not only obtain the preference of others for the speedy advancements that might be made in the Art of Physick, but likewise encourage *English* men and Foreigners to spend their time amongst us, the advantages and improvements being so much greater here, than elsewhere to be obtain’d. (Ibid., 61)

In effect, Goodall, building on the earlier proposals, was sketching the system of clinical education including morbid anatomy which emerged in London during the eighteenth century.

Although they differed in details, particularly in their relative focus on learning from working with an experienced practitioner or from the hospitals, these controversialists all argued for establishing a new, London-based, period of practical and vocational training which could match the opportunities offered on the Continent and replace the need for newly-qualified practitioners to return home with only their scholastic knowledge to learn how to practice gradually thereafter. In doing so they would create a fusion of theoretical and practical experience superior to what their rivals, the apothecaries, could offer through apprenticeship.

## 5. The Status of Apprenticeship

The notion of younger doctors being associated with their seniors for a period of training (whether in individual practice or in hospitals) inevitably raised the model of “apprenticeship.” Some critics of the College during the Restoration debate advocated that a full apprenticeship model should be adopted and even proposed that such training should be brought, like other apprenticeships, under the regulatory authority of the city government. The physician Marchamont Nedham, comparing the skills and utility of physicians unfavorably with that of surgeons, argued

that the Laboratory, the Work-house is the way to be traced before we enter the Library; an Apprenticeship from our Youth to work and study under a Practiser, is that only which can make one a Doctor … and our Youth for Physick, instead of being *Academians*, be bred up *more Mechanico*, instituted in the Operative, before they bend themselves upon the Contemplative and Philosophizing part of Physick.

Nedham hoped that

the Magistrates, Nobility and Gentry of the Land, will accordingly steer their Judgment in the choice of Physicians, and one time or other give a helping hand towards a reforming of the Education of Men for the practice of this Faculty.

He prioritized

the most worthy *Art of Chirurgery:* which as it is the most Antient of all the Parts of Medicin, so, next after the Accomplishments of a Grammar-School, it ought to be the first thing that he who aims to be a Physician, should propose unto himself, and accordingly serve seven years therein to some Master, that is able to tutor him in the daily practice of it, and of Chymical Operations, and of Curing Diseases, as the only Method of Education, out of the common Road of an University, to bring a Man to be *indeed a Doctor.* (Nedham 1675, “To the Reader” [unpaginated])

The apothecary turned physician Adrian Huyberts also criticized the “great Clamor by the *Collegiates*, against many ingenious men [such as himself], whose first Education in the world was not in this Art, but afterwards betook themselves to learn it in the most proper way of learning; which is by labor, and have soon out-strip’t the Scholasticks in right knowledge of the *Materia Medica*” (Huyberts 1675, 28). During his lifetime Huyberts had seen “the old Education in Academies judged incompetent, the places themselves being too narrow to afford much observation or experience, and the manner of life more speculative and notional than Mechanick or laborious, which a Physitian’s ought to be.” Academic learning could only “fit him to be bound Prentice to some able Practiser in a populous City, that under him he may work out his way, to become *indeed a Doctor*” (ibid., 11). But

if the whole Body of Physicians here in this City be really the Physicians of *London*, Why may they not, being part of the City, be taken hereafter under the City Government? Be obliged to take Apprentices, such young men as have taken degrees in other Arts at some University; who when they have served their time at work under a City-Physician, may then be made a free Practiser of *London?*

Appealing to the government, Huyberts maintained that

such a populous City is the only place (being a Theatre of all Diseases) wherein to breed up men Physicians indeed; such as may practise with real knowledge; not fill the world with Cobwebs of idle speculations and notions, as men of the old way of Education are wont to do; and which may furnish his Majesties Armies and Fleets Royal, with Physicians, as the Society of Chirurgerie do with Chirurgians. (Ibid., 27-8)

But for the supporters of the London College, civic apprenticeship, as proposed by their critics, would have been unacceptable: the whole point was to establish collegial self-regulation, managed by the senior members of the faculty. Even so, they faced the concern that being “apprenticed” was unsuitable for the education of a gentleman, implying servile manual labor, and so undermining the distinctive image of the physician (Pelling 2019). T.M. addressed this directly, as usual seeking to offer a conservative justification for the change of practice:

Nor let any man think to disgrace this Method as Mechanical, by the imputation of taking Apprentices; since the word Apprentice is entertained by the honourable Profession of the Law, whose younger Students have been call’d Apprentices to the Law; but however this be, it were very fond for so poor an occasion, to neglect a thing that is founded upon the evidence of true reason itself. Nor indeed should they be received in the capacity of Servants, or under that name, but rather of young *Students, Friends*, or *vertuous Companions* to be instructed in this worthy Profession; the drudgery of all things resting wholly upon some ordinary Servant kept by every one for the uses of his Family: and I make no doubt but there are very many ingenious young men in *England*, who would be very glad, and take it for a great honour, to be thus received by some of the Grandees, and great Practitioners; and their Friends believe whatever is bestowed on them in this way very well and honourably employed. (T. M. 1665, 33-4)

If it was already good enough for lawyers, and provided that it was seen as a collaboration of equals, not a servant/master relationship, then an apprenticeship model could be adopted without dishonor. Indeed, the senior/junior relationship that was being proposed might be seen as modelled as much on a father/son model as a master/apprentice one – an analogy regularly used in describing how some practitioners trained and passed on their practice, and quite often, of course, literally the case, as practitioners would educate one or more of their sons (or even their daughters) to follow them. Physicians’ children (if they had any – Margaret Pelling has pointed out how often physicians married late, or never) had, of course, a considerable advantage in what was still a household economy of practice, and inheritance, like apprenticeship (itself a surrogate parenthood), was still a key mode of transmission of knowledge (Pelling 1995b and 2005). As we have seen, despite all the emphasis on academic learning, international travel or even experimental expertise, the great majority of provincial physicians returned to practice in their home town or nearby, because establishing a practice owed more to networks of kinship, patronage, and credit than it did to formal qualifications or technical expertise. However, in London (already almost thirty times the size of any other English town) such informal relationships (though very often no doubt still significant) were already inadequate to establish a secure basis for trust. But it was only during the eighteenth century, when the traditional model of establishing a practice began to crumble in other, rapidly-growing towns, and with the rise of spas, seaside resorts, and practice in the service of the state, all involving increased mobility of practitioners to places where they had to rely solely on their professional qualification to establish that they had been properly trained in both the theory and practice of medicine, that these reforming ideas would be converted (at least in part) into reality.

## 6. Conclusion

There is no sign that the London College of Physicians as an institution ever seriously explored the possibility of putting these reform proposals into practice, and there were no further significant proposals along these lines after 1676, even though the broader medical disputes continued to rage unabated in London. Indeed, the institutional conflicts which continued into the early eighteenth century probably ensured that no planned public reforms could have won the consensus needed for them to take place. Instead, they stand as an interesting blueprint for the model of medical education which started to emerge in eighteenth-century London, and was then gradually made a statutory requirement by various stages in the nineteenth century. Unlike in Scotland, where an alliance of medical men, civic leaders, and aristocrats built on a growing body of private initiatives and institutionalized them, with spectacular success, at Edinburgh University (Dingwall 2010; Emerson 1993 and 2004; Risse 1987-8; Rosner 1991), in England a new approach arose piecemeal from private undertakings, and was not transformed into a state-supported educational model until after 1800.^6^ Many other historians have traced this process, although relatively little attention has been paid to the period before the 1730s, when it began to gather momentum, and there is insufficient space here to do more than sketch some features.

As noted above, it looks as if medical men in London’s three state hospitals (Barts, St Thomas, and Bethlem) began to offer trainees (for fees) the chance to work with them on the wards after 1660, although without official approval (Waddington 2003; Wilson 1992). This practice not only grew in these institutions, but also became established from the start in the many charitable and voluntary hospitals established in London early in the eighteenth century and in the provinces from the mid-1730s onwards, although these “teaching” arrangements remained essentially private, being seen as part of the perquisites of the post, especially for those who gave their medical services free (or, if salaried, for much less than they could earn privately). An increasingly important advantage of becoming hospital pupils was not just clinical experience in the wards, but also access to dissections, although the lay managers of hospitals remained reluctant to see patients treated as guinea-pigs, and were very sensitive to the use of corpses, even of the poor.

Partly for this reason, there grew up alongside the hospitals from the 1690s a network of private lectures and eventually courses, run mostly by surgeons (some also practicing as physicians), where not only students but also an interested public could learn more about the body and disease, but which in some cases came to offer structured anatomical lessons and even the opportunity for students to get hands-on experience of dissection themselves (as well as a close-up view of dissection done by the teacher). A major drive in this was the growth of male-midwifery as a key attribute of the urban medical practitioner, and the increased naval and military demand for surgical skills. It also reflected (and strengthened) the focus on morbid anatomy we saw presaged by Clarke and Goodall.

It seems likely that these developments originally affected only those based in London. But, by the 1730s at least, provincial medical trainees, perhaps after a conventional local apprenticeship in surgery or pharmacy, began to regard a period in London attending such courses and visiting the wards as a necessary stage in completing their education, if they could not afford a period abroad (where Paris was supplanting Leiden as the place of choice) or in Edinburgh. Gradually, as Goodall had predicted could happen, London became not only an acceptable destination for English students, but even attracted Scottish and continental students to study with such luminaries as the Hunter brothers. Late in the century, as provincial towns and their hospital opportunities expanded, some of the major centers like Birmingham, Manchester, and Bristol began to develop similar programs, although it was hard for them to compete with London or Edinburgh in prestige. They also sought to develop a “collegial” setting by forming medical societies that were often based on a library, to remind us of the continuing role of book learning, although the books that many practitioners were now publishing were increasingly reports of their medical practice (Reinarz 2007, 2008, 2009).

However, in several important respects, what developed was far from what the Collegians had intended, even if it remedied the defects in previous education which they had identified. Not only did the College of Physicians as an institution play no part in these educational developments, neither did physicians as an occupational group. Instead, they were largely driven by the growing prestige of both surgery and pharmacy, as the general practitioner who was surgeon, apothecary, and male-midwife (sometimes also with a Scottish M.D.) became the standard of medical training, at the expense of the traditional physician (Burnby 1995; Loudon 1986). Moreover, the inability of the English state to create a London university until the 1820s meant that these developments took place outside a university environment, with Oxford and Cambridge far more isolated than they had been in the 1650s and 1660s (Singer and Holloway 1960). Hence, when medicine was first brought into a national framework of education and qualification in the nineteenth century, it was under the aegis of the Society of Apothecaries and through the spread of teaching schools in hospitals, not through the combination of university education and Collegiate informal apprenticeship proposed by the Restoration physicians (Holloway 1966; Loudon 1994; van Zwanenberg 1983). The union of medical minds and hands, in England at least, was modelled on the surgeon/apothecary (or the general practitioner), not on the collegiate physician.

## Notes

1 The historiography to date is summated in Nutton and Porter 1995. This article does not address directly the education of female medics (including midwives), though its findings about the importance of place, family, and non-formal education help to explain why female medical expertise was valued (Evenden 1998 and 2000, chaps. 1-2; King 1995; Pelling 1995a).

2 Krivatsky 1975; and Wilson 1999, chap. 2, are rare among studies of ordinary medical practitioners in considering training in depth: cf. Beier 1987, chaps. 3 and 4, which contain very little on Binns’ or Symcott’s education.

3 See for example Harvey 1670, 17-26, and 1678, 140-3, the first describing an ideal education and the second Gabriel Harvey’s actual education, which follows precisely the same pattern.

4 This section summarizes the arguments developed in Barry 2018.

5 For a bibliography of the debate see Cook 1989, 169-71 and for *A Letter* Cook 1986, 141-3. The (much-debated) authorship of this text is attributed to Dr. William Croone (1633-84) in Cooper 1673. An alternative author proposed by Peter Elmer is (the future Sir) Thomas Millington (1628–1704), then based in Oxford, who has the correct initials and would have been writing from outside London, as the letter-writer claims. George Castle, *The Chymical Galenist* (London, 1667) was dedicated to Millington who had seen a draft early in 1665, roughly when “T.M.” published his *Letter* (imprimatur 30 March).

6 The most informative single account is Bynum 1985, along with other essays in Bynum and Porter 1985, but see also Lawrence 1988 and 1996.
